# Settlement Laws and Public Institutions

**Published:** 1905-08-12

**Authors:** 


### SETTLEMENT LAWS AND PUBLIC INSTITUTIONS.

The laws of settlement have been a trouble ever since they were enacted, and probably it is impossible wholly to avoid injustice in connection with them. When birth or a residence of a certain fixed period gives a settlement in a parish, the ends of justice—in making each locality bear its own burdens—will, on the whole, be sufficiently well served. But anomalies and injustice occasionally occur, and it might be well if the kind of residence needful to acquire settlement were more elaborately defined. A pauper does not acquire a settlement in a parish through his residence in it, if at the time he became a pauper his settlement was in another parish. Nor does a lunatic acquire settlement in a parish through residing in an asylum within its boundaries. This is obviously reasonable, for settlement should be earned by work or rate-paying for a certain time within the parish limits, and if paupers or lunatics could acquire a settlement, it is clear no parish would allow any one to remain who was in receipt of non-settled relief, and this would bear hardly on many old people who are living with children outside their own parish. While, as to lunatics, it would be difficult to obtain a site for an asylum if there was the possibility that many of the occupants would one day come upon the local rates. But the same rules are not applied to charitable institutions generally. Thus a child brought up in a home within the union for four years acquires thereby a settlement in that union, and if his health fails before he has acquired a settlement in any other union, the ratepayers of the one where the orphanage or other institution which he was brought up is situated are liable to keep him for life. Happily there are comparatively few cases in which this claim is acted upon, for if they were numerous and the thing became known it would be almost impossible for the promoters of any charitable institution to obtain ground on which to build it, unless, indeed, they gave an undertaking that none of the inmates would ever become chargeable to the parish. But it is obviously reasonable that, failing a settlement acquired by residence with rate-paying relatives, a birth settlement should hold good for a child until he himself becomes a wage-earner. The status of a hospital patient with regard to settlement is still a matter of dispute. Some authorities hold that a patient dying in a hospital outside his own parish is "constructively" still a resident in the parish where he lived before he entered the hospital—as a sailor is "constructively" regarded as living in the parish where he has a home and pays rates. Others hold that his status is lost when he goes into the hospital. This does not of course affect the patient himself, but it affects his widow, who may lose her status of irremovability, and therefore be refused help by the parish in which she has been living. A minor grievance occurs where there is a prison in a union. If a prisoner is ill when his sentence is completed, the governor of the gaol may send him to the infirmary of the union where the prison is situated, though he has no real claim to assistance there. In some cases the prisoner is sent back to the place where he was convicted, and no objection is made to this course. It is, indeed, the reasonable thing to do, but it is not strictly legal, and no prison governor can be compelled to follow this course. If such details were clearly decided once for all, it would save much trouble and occasional irritation.

